# Biological effects of electron beam to target turning X-ray (EBTTX) on two freesia (*Freesia hybrida*) cultivars

**DOI:** 10.7717/peerj.10742

**Published:** 2021-01-28

**Authors:** Yi-rui Li, Ling Liu, Dan Wang, Li Chen, Hao Chen

**Affiliations:** 1 College of Life Science and Engineering, Southwest University of Science and Technology, Mianyang, China; 2 State Key Laboratory of Grassland Agro-ecosystem, Lanzhou University, Lanzhou, China; 3 College of Architecture and Environment, Sichuan University, Chengdu, China; 4 Sichuan Institute of Atomic Energy, Chengdu, China

**Keywords:** Mutation breeding, Electron beam to target turning X-ray, Freesia hybrida, Plant growth and development, Micro-morphology

## Abstract

Electron beam to target turning X-ray (EBTTX) is an emerging irradiation technology that can potentially accelerate the breeding process of plants. The biological effects of EBTTX irradiation on the two freesia cultivars (the red freesia and the purple freesia) were investigated by establishing an irradiation-mediated mutation breeding protocol. The germination rate, survival rate, plant height, leaf number and area, root number and length of the two freesia cultivars decreased following different irradiation doses (25, 50, 75, and 100-Gy). A high irradiation dose exhibited stronger inhibition effects on these plant growth parameters, and the survival rate of the two freesia cultivars was 0.00% following the 100-Gy irradiation treatment. The median lethal dose (LD_50_) based on survival rates was 54.28-Gy for the red freesia and 60.11-Gy for the purple freesia. The flowering rate, flower number, and pollen vigor were significantly decreased by irradiation treatment. At 75-Gy irradiation, the flowering rate, flower number and pollen viability of the two varieties reached the minimum, exhibiting strong inhibitory effects. Meanwhile, 75-Gy irradiation significantly decreased the chlorophyll content and increased the malondialdehyde (MDA) content of the two freesia cultivars. Furthermore, as the irradiation dose increased, the changes in the micro-morphology of the leaf epidermis and pollen gradually increased according to a scanning electron microscope (SEM) analysis. These results are expected to provide useful information for the mutation breeding of different freesia cultivars and other flowering plants.

## Introduction

Freesia (*Freesia hybrida*), an herbaceous ornamental plant, was first found growing in southern Africa (Gao et al., 2018). As a cut flower, it is a famous winter houseplant due to its varied colour, long flowering period, and pleasing fragrance. Meanwhile, freesia is also one of the top ten cut flowers in the world and has an important role in the field of international flowers (Huang et al., 2018). Freesia does not only have high ornamental value but also its fragrance has the effects of inhibiting sympathetic nervous excitement and reducing blood pressure (Sun et al., 2016). After many years of cultivation, the varieties of freesia seriously degraded, which has led to some disadvantages in large-scale practice such as small inflorescence, short stem, and the aggravation of virus damage (Li et al., 2019). Therefore, it is of great concern and has become a research hotspot to breed new cultivated varieties of freesia and improve the quality of freesia varieties.

Several strategies have been used for enhancing seed breeding in plants, such as cross breeding and selective breeding (Zhang et al., 2014; Vleeshouwers & Oliver, 2014). However, these strategies have some disadvantages including heavy workload and decrease in fertility of the offspring (Yamaguchi, 2018). Mutation breeding, especially breeding induced by ionizing radiation, has been used as an effective tool to obtain new plant varieties of agronomic interest (Yamaguchi, 2018; Kim et al., 2019). Mutation breeding induced by ionizing radiation has been proven to increase plant chromosomal variation, cause changes in morphology, physiology, and biochemistry, and breed new varieties in a short cycle (Su et al., 2019). Meanwhile, mutation breeding by ionizing radiation can also be widely used in the quality improvement of flowering plants (Yu et al., 2016). The phenotypes of these new flower varieties show many characteristics such as varied colour, shape, size, and flowering period (Yu et al., 2016; Yamaguchi, 2018). Several studies have focused on the biological effects of ionizing radiation on ornamental plant seeds. Yu et al. (2016) confirmed experimentally that carbon ion beam irradiation is effective in inducing genomic variations of geranium (*Pelargonium* ×* hortorum*, Bailey), which induces changes in the flower colour and anthocyanin accumulation in the plant. Kim et al. (2019) reported 25-Gy gamma irradiation facilitated the growth of creeping bentgrass, which increased the shoot length, shoot width, root length, leaf blade length, sheath length, and fresh weight compared with control groups. Li et al. (2019) also demonstrated that a low dose (25 Gy) of X-ray radiation could promote the growth and development of Gladiolus gandavensis, whereas a high dose (100 Gy) of X-ray radiation significantly inhibited plant germination and growth.

Leaf chlorophyll is the major pigment produced in chloroplasts in plant cells, which can significantly affect the physiology, photosynthetic intensity of plants, and leaf colour formation. Meanwhile, malondialdehyde (MDA) is a final decomposition product of lipid peroxidation and is considered a major indirect indicator of abiotic stress-induced oxidative damage (Chen et al., 2021). To date, mutation breeding has been widely used in ornamental plants. Meanwhile, several researchers reported that ionizing radiation can cause huge damage to the growth of ornamental plants such as *Gladiolus gandavensis* (Li et al., 2019), *Cerasus* sp. (An et al., 2020), and *Cymbidium* ssp. (Kim et al., 2020).

Electron beam to target turning X-ray (EBTTX), a new type of irradiation technology, is a bremsstrahlung X-ray produced by the sudden deceleration of a high-energy electron beam bombarding heavy metals. EBTTX was not only used to sterilize the fungal and bacterial colonies on various material surface, but also can provide variation in plant structure and function from which breeders can select plants having useful traits (Borgognoni et al., 2017; Saha & Paul, 2019). This irradiation technology has the advantages of both electron beam and X-ray irradiations such as easy operation, high accuracy, no radioactive source, strong penetrating ability, and good repeatability (Orazem, Štajner & Bohanec, 2013; Mondal et al., 2017). Moreover, the irradiation technology can shorten the cycle of mutation breeding and improve the efficiency of mutation breeding due to that the tested plants undergo double mutation under the irradiation treatments. Currently, EBTTX has been widely used in the fields of food, medicine, physics, and materials, etc., but it is rarely reported in the breeding of ornamental plants. (Gautam & Tripathi, 2016; Chauhan & Wilkins, 2018). Therefore, we hypothesise that EBTTX radiation has the potential to breed new freesia varieties by altering the morphological and physiological parameters of the plants. The objectives of this study were: (1) to explore a new irradiation breeding method (EBTTX) for the two freesia varieties; (2) to investigate the radiation biological effects of EBTTX on the two freesia varieties; (3) to analyse preliminarily the micro-morphology of leaf and pollen irradiated by EBTTX; and (4) to establish the LD_50_ dose model under EBTTX. The results of this study benefit the evaluation of the effects of irradiation treatments on the biological effects of freesia and can provide a theoretical basis for mutation breeding of freesia cultivars.

## Materials & Methods

### Plant material

Two freesia cultivars, *F. armstrongii* (the red freesia) and *F. corymbosa* (the purple freesia), were purchased from the Sichuan Deyang Oriental Flower Co., Ltd. Approximately same size 300 healthy bulbs were purchased for the both freesia cultivar. Meanwhile, the average weight of the bulbs was 2.22 ± 0.19 g for the red freesia and 2.28 ± 0.16 g for the purple freesia. The average diameter of the bulbs was 3.58 ± 0.22 cm for the red freesia and 3.42 ± 0.17 cm for the purple freesia.

### Irradiation conditions

The bulbs of the two freesia varieties were irradiated by electron beam to target turning X-ray (D/Max-RB, Rigaku Corporation, Tokyo, Japan) at the Sichuan Institute of Atomic Energy (Chengdu, Sichuan province) at dosages of 0 (CK, control), 25, 50, 75 and 100-Gy, and the energy and the flow rate were 2 MeV and 10 mA, respectively (September 8, 2017). Triplicate repetitions per treatment were conducted, and 20 bulbs in each repetition were involved.

### Plant cultivation

A pot experiment was conducted in a greenhouse (10–25 °C) at the Southwest University of Science and Technology (Mianyang, Sichuan Province). The irradiated bulbs were planted into flowerpots (L × W × H = 49 cm ×20 cm ×18 cm) in the greenhouse, for a total of 20 bulbs per flowerpot (September 10, 2017). The nutrient soil is composed of special plant cultivated soil, fermentative organic substrate, leaf mould, and vermiculite, and the volume ratio is 5: 2: 2: 1. Furthermore, all flowerpots were arranged according to a complete randomized design (triplicate), and the soil was regularly irrigated with tap water.

### Measurement methods

### Plant growth and development parameters

All parameters are counted every 10 days. The germination rate and survival rate was measured at 10 to 20 days and 30 to 80 days after cultivation, respectively (Table 1). The plant height and number of leaves were recorded at 10 to 80 days after cultivation. The leaf area, root length and number were recorded at the 80th day (Table 1). Leaf area is the maximum leaf area of each plant, and root length is the longest root length of each plant. Furthermore, median lethal dose (LD_50_) of the tested plant was determined according to the method of Huang et al. (2016). In this study, EBTTX radiation dose is considered the abscissa, and the survival rate is the ordinate of the fitting linear equation (*y* = *ax* + *b*). Median lethal dose is calculated according to the linear equation when the lethality rate was 50%.

**Table 1:** The recording time of different morphological indexes.

Morphological indexes	Recording time
Germination rate-1	September 20, 2017
Germination rate-2	September 30, 2017
Survival rate-1	October 10, 2017
Survival rate-2	October 20, 2017
Survival rate-3	October 30, 2017
Survival rate-4	November 9, 2017
Survival rate-5	November 19, 2017
Survival rate-6	November 29, 2017
Plant height and number of leaves-1	September 20, 2017
Plant height and number of leaves-2	September 30, 2017
Plant height and number of leaves-3	October 10, 2017
Plant height and number of leaves-4	October 20, 2017
Plant height and number of leaves-5	October 30, 2017
Plant height and number of leaves-6	November 9, 2017
Plant height and number of leaves-7	November 19, 2017
Plant height and number of leaves-8	November 29, 2017
The leaf area, root length and number	November 29, 2017

After 180 days of cultivation (March 27, 2018), the flowering rate, pollen vigour, and number of flowers were measured. The pollen vigour was detected by the 2, 3, 5-triphenyltetrazolium chloride (TTC) staining method according to the methods of Soares et al. (2015). Meanwhile, the chlorophyll contents of fresh leaves were determined as follows. After extracting 0.25 g of fresh leaves of the two freesia varieties in an 80% aqueous acetone, the absorbance of the filtrates was determined with an ultraviolet spectrophotometer (NanoDrop 2000; Beijing Purkinje General Instrument Co., Ltd, Beijing, China) at the following wavelengths: 665 nm for chlorophyll a and 649 nm for chlorophyll b. Total chlorophyll content was calculated using the method of Yue et al. (2009). Furthermore, the MDA content of fresh leaves was determined with the thiobarbituric acid reaction according to the methods of Saidi, Chtourou & Djebali (2014).

### Pollen grain and leaf surface microstructure

Fresh pollen was collected in a centrifuge tube during the pollinating period and placed in a drying oven for 12 h. The pollen was smeared on a double-sided adhesive surface on a copper stage and sent to a vacuum coating machine for 2 min, then observed directly by scanning electron microscope (EVO18, Carl Zeiss, Jena, Germany). Furthermore, the same part (2  mm ×2 mm) in the leaf mid position was cut out, dehydrated 3 times with 100% ethanol, soaked in 100% tert-butanol for 15 min, and then placed in a drying oven for 12 h. The leaf was smeared on a double-sided adhesive surface on a copper stage, sent to a vacuum coating machine for 3 min, and then observed directly by SEM.

### Computational method

Seedling rate = (number of bulb germination/number of sow bulbs) ×100%

Survival rate = (number of surviving plants/number of sow bulbs) ×100%

Flowering rate = (number of flowering plants/total number of plants) ×100%

Pollen viability per plant = (vibrant pollen/total number of pollen) ×100%

### Statistical analysis

All data were analysed using Excel 2010 and SPSS 23.0 to determine the least-significant difference (LSD) between different treatments (*p* < 0.05). Figures were drawn by Origin 2018b.

The results are expressed as the mean ± standard deviation (triplicate).

## Results

### Effects of different irradiation doses on plant growth parameters of two freesia varieties

#### Germination rate

Effects of different irradiation doses on the germination rates of the two freesia varieties are shown in Table 2. As irradiation dose increased, the germination rates of the two freesia varieties decreased. Meanwhile, the bulbs of the two freesia varieties could not germinate following with high irradiation doses (75 or 100-Gy) during the first 20 days of cultivation. Under the 50-Gy treatment, the germination rates of the purple freesia were higher than those of the red freesia.

**Table 2:** Influences of different irradiation doses on germination rates of two freesia varieties.

Varieties	Time (day)	Germination rate (%)				
		0 (CK)	25 Gy	50 Gy	75 Gy	100 Gy
Red freesia	10	45.00 ± 5.00a	35.00 ± 5.00a	1.67 ± 2.89b	0.0 ± 0.0b	0.0 ± 0.0b
	20	73.33 ± 12.58a	66.7 ± 10.4b	18.33 ± 2.89c	0.0 ± 0.0c	0.0 ± 0.0c
Purple freesia	10	51.66 ± 10.41a	43.33 ± 5.77b	16.67 ± 2.89c	0.0 ± 0.0d	0.0 ± 0.0d
	20	68.33 ± 7.64a	63.33 ± 10.41a	45.00 ± 8.66b	0.0 ± 0.0c	0.0 ± 0.0c

**Notes.**

All data are expressed as the mean ± standard deviation. Lowercase letters on the same line represent statistically significant differences at *p* < 0.05.

#### Survival rate

The survival rates of the two freesia varieties decreased gradually with increasing irradiation dose (Table 3). Meanwhile, the survival rates of the two freesia varieties were 0.00% following 100-Gy treatment during 30 to 80 days. Furthermore, the purple freesia exhibited a lower downward trend for the survival rate following different irradiation doses compared with the red freesia.

**Table 3:** Influences of different irradiation dose on survival rates of the two freesia varieties at 30 to 80 days.

Varieties	Dose (Gy)	Survival rate (%)					
		30 days	40 days	50 days	60 days	70 days	80 days
Red freesia	0	83.33 ±11.55^a^	93.33 ±7.64^a^	95.00 ±8.66^a^	95.00 ±8.66^a^	95.00 ±8.66^a^	95.00 ±8.66^a^
	25	80.00 ±5.00^a^	88.33 ±12.58^a^	88.33 ±12.58^b^	88.33 ±12.58^ab^	88.3 ±12.58^ab^	88.33 ±12.58^ab^
	50	40.0 ±8.66^b^	58.33 ±7.64^b^	73.33 ±10.41^c^	73.33 ±10.41^b^	75.00 ±8.66^b^	75.00 ±8.66^b^
	75	1.67 ±2.89^c^	1.67 ±2.89^c^	3.33 ±2.89^d^	3.33 ±2.89^c^	3.33 ±2.89^c^	3.33 ±2.89^c^
	100	0.0^c^	0.0^c^	0.0^d^	0.0^c^	0.00^c^	0.0^c^
Purple freesia	0	83.33 ±7.64^a^	86.67 ±5.77^a^	88.33 ±7.64^a^	88.33 ±7.64^a^	88.33 ±7.64^a^	88.33 ±7.64^a^
	25	78.33 ±12.58^ab^	81.67 ±12.58^ab^	83.33 ±10.41^ab^	83.33 ±10.41^a^	83.33 ±10.41^a^	83.33 ±10.41^a^
	50	71.67 ±12.58^b^	71.67 ±12.58^b^	73.33 ±10.41^b^	78.33 ±10.41^a^	81.67 ±7.64^a^	81.67 ±7.64^a^
	75	8.33 ±2.89^c^	13.33 ±5.77^c^	16.67 ±2.89^c^	16.67 ±2.89^b^	16.67 ±2.89^b^	16.67 ±2.89^b^
	100	0.0^c^	0.0^d^	0.0^d^	0.0^c^	0.0^c^	0.0^c^

**Notes.**

Lowercase letters on the same column represent statistically significant differences at *p* < 0.05.

### Median lethal dose (LD_50_) analysis

The equation of linear regression between the survival rates (80th day) and the irradiation doses was *Y* =  − 1.10*x* + 107.33 (R^2^ = 0.86) for the red freesia and *Y* =  − 0.97*x* + 102.66 (R^2^ = 0.83) for the purple freesia (Table 4). The results reveal a positive correlation between irradiation dose and lethality rate of the two freesia varieties. Meanwhile, the LD_50_ was 54.28-Gy for the red freesia and 60.11-Gy for the purple freesia.

**Table 4:** Survival rate, median lethal dose (LD_50_), and correlation coefficient of the two freesia varieties.

Varieties	Survival rate (%)					LD_50_ (Gy)	Correlation coefficient
	0 (CK)	25 Gy	50 Gy	75 Gy	100 Gy		
Red freesia	95.00 ±8.66^a^	88.33 ±12.58^ab^	75.08 ±8.66^b^	3.33 ±2.89^c^	0.0^c^	54.28	0.86
Purple freesia	88.33 ±7.64^a^	83.33 ±10.41^a^	81.67 ±7.64^a^	16.67 ±2.89^b^	0.0^c^	60.11	0.83

### Plant height

**Figure 1:**
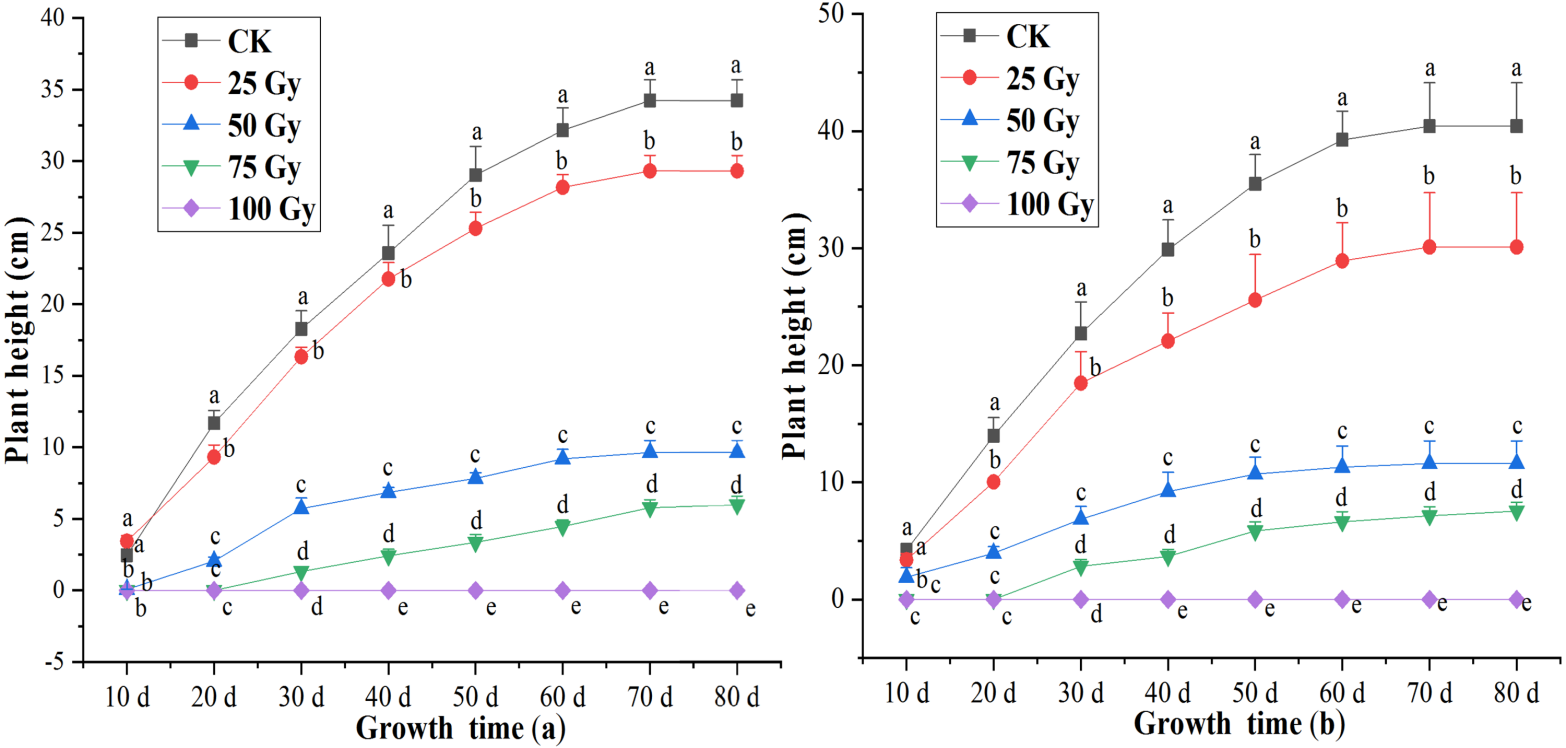
Influences of different irradiation dose on plant height of the rea freesia (A) and the purple freesia (B). Lowercase letters on the same column represent statistically significant differences at *p* < 0.05. Error bars indicate the standard error of the mean for *N* = 3 independent experiments.

A significant difference (*p* < 0.05) in plant height was observed between controls and plants irradiated with different irradiation doses during 20 to 80 days after cultivation (Fig. 1). Inhibition effects of EBTTX irradiation on plant height significantly (*p* < 0.05) enhanced with increasing irradiation dose. The shoots of the two freesia varieties could not grow following the 100-Gy treatment. Under 75-Gy treatment, the two freesia varieties were observed to germinate until 40 days, and no significant difference detected in the plant height after 60 days. The maximum decline of plant height was 82.54% for the red freesia and 81.34% for the purple freesia, respectively, and was observed at 75-Gy at 80 d after cultivation.

### Leaf number, leaf area, root number and length

**Figure 2:**
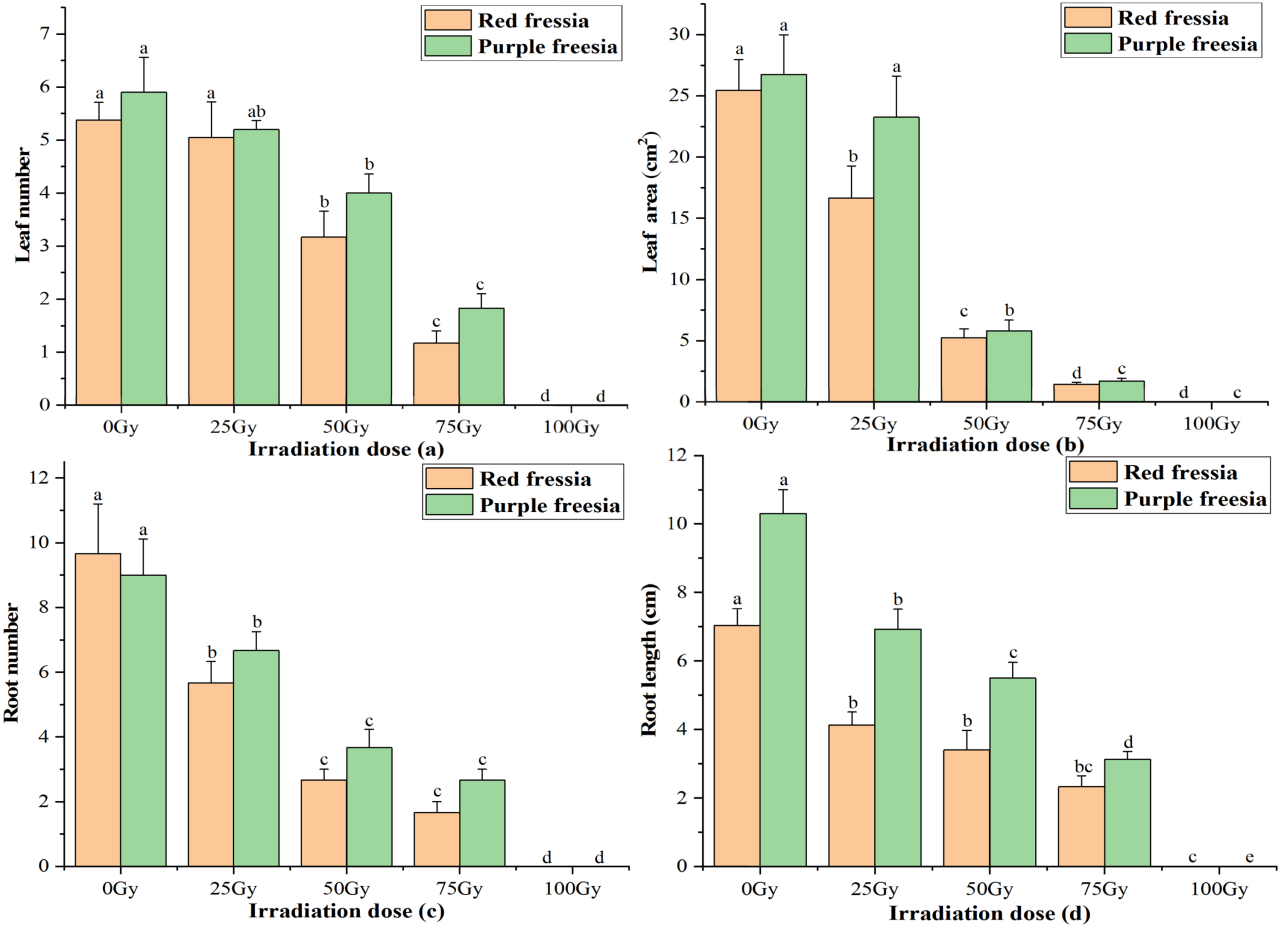
Influences of different irradiation dose on leaf number (A), leaf area (B), root number (C) and length (D) of the two freesia varieties. Lowercase letters on the same freesia variety represent statistically significant differences at *p* < 0.05. Error bars indicate the standard error of the mean for *N* = 3 independent experiments.

The leaf number and area of the two freesia varieties were significantly affected by EBTTX irradiation, and this effect was dependent on the irradiation dose applied (Fig. 2). The leaf number and area of the two freesia varieties decreased with increasing irradiation dose. Meanwhile, the leaf number and area were significantly (*p* < 0.05) inhibited compared with the control when the irradiation dose was greater than or equal to 50-Gy. In particular, with 100-Gy treatment, the shoots of the two freesia varieties could not even grow due to strong irradiation toxicity.

For root number and length of the two freesia varieties, all EBTTX irradiation doses resulted in a significant (*p* < 0.05) decrease compared with controls. Meanwhile, the root number and length were further decreased with increasing irradiation dose. Interestingly, the root number of the red freesia in the control was higher than that of the purple freesia, but the purple freesia under different irradiation doses (except 100-Gy) had higher root numbers than the purple freesia.

### Flowering rate, flower number, and pollen vigour

**Figure 3:**
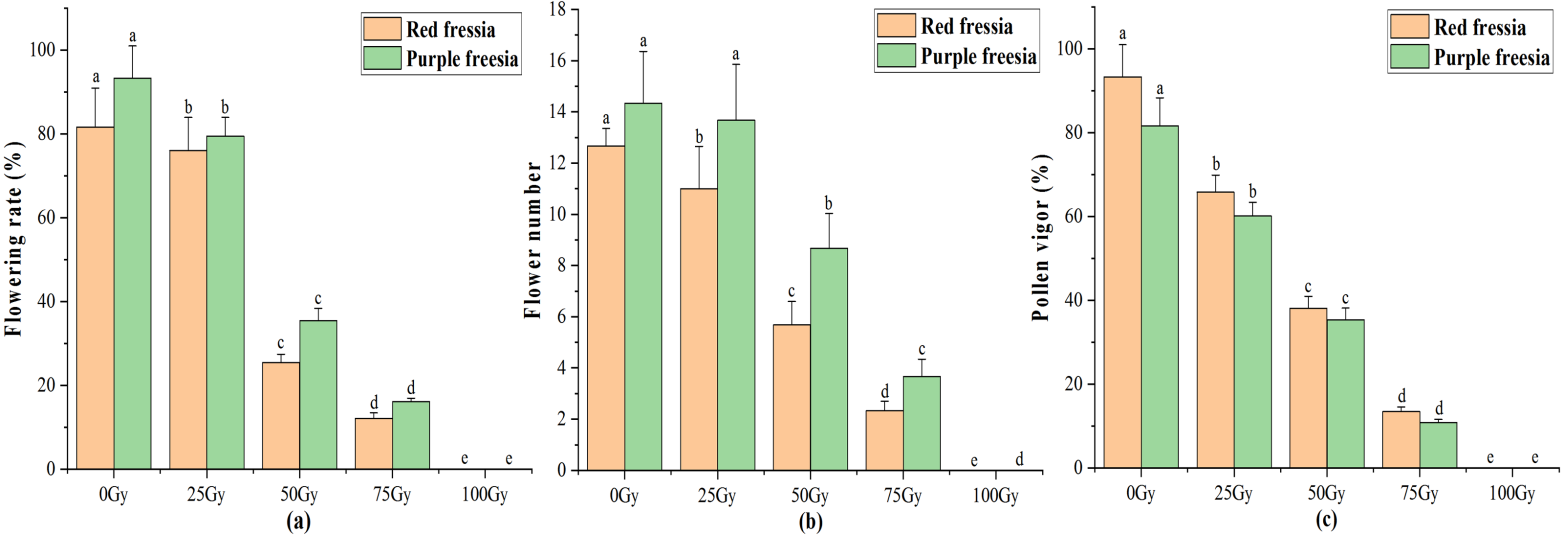
Influences of different radiation doses on flowering rate (A), flower number (B), and pollen vigor (C) of the two freesia varieties. Lowercase letters on the same freesia variety represent statistically significant differences at *p* < 0.05. Error bars indicate the standard error of the mean for *N* = 3 independent experiments.

The application of the EBTTX irradiation significantly (*p* < 0.05) decreased the flowering rate of the two freesia varieties (Fig. 3A). Under 75-Gy treatment, the flowering rate was 12.13% for the red freesia and 16.11% for the purple freesia, which was 69.54% and 77.22% lower than that of the control, respectively. Meanwhile, the flower number and pollen vigour continuously decreased with increasing irradiation dose (Figs. 3B and 3C). The pollen vigour was 13.44% for the red freesia and 10.78% for the purple freesia at 75-Gy irradiation treatment, which was decreased by 79.89% and 70.89%, respectively, compared to controls.

### Effects of different radiation doses on total chlorophyll content and MDA of the two freesia varieties

**Figure 4:**
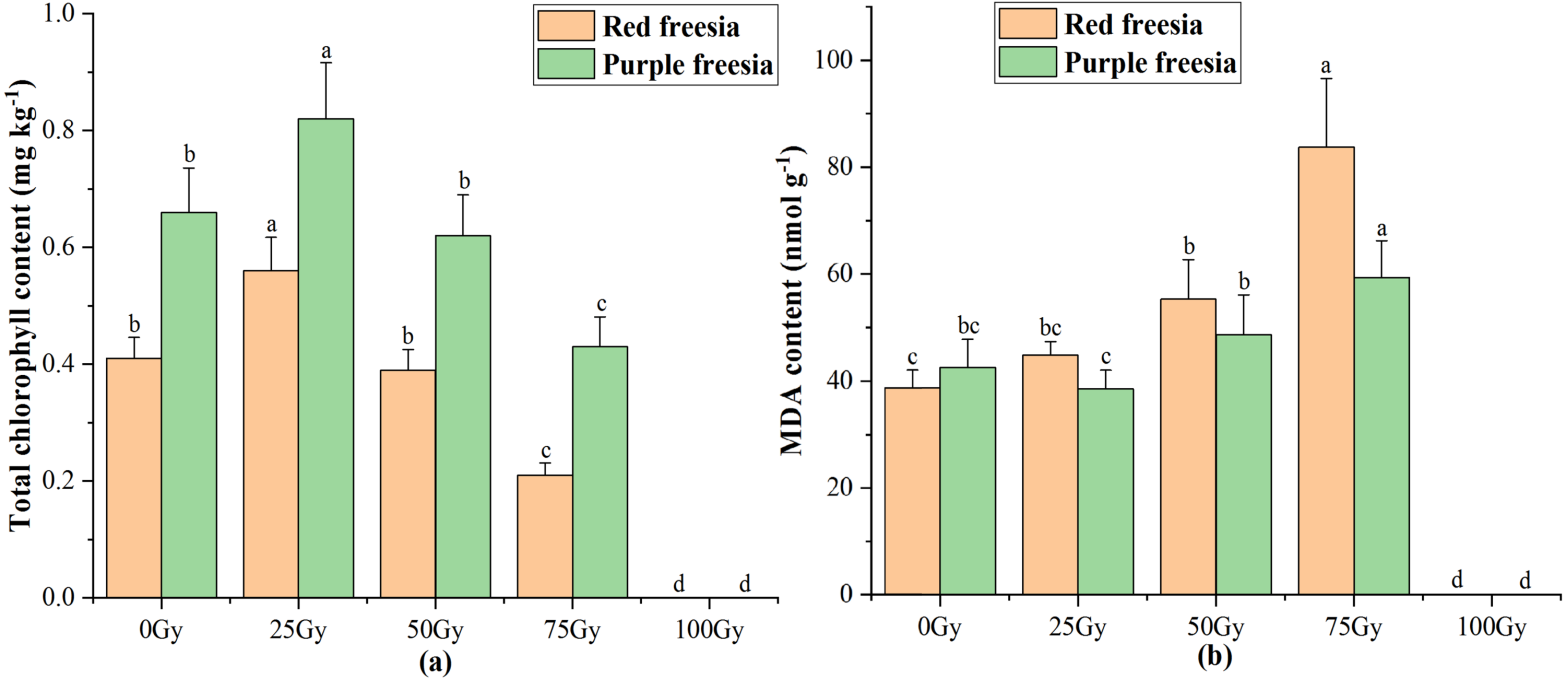
Influences of different irradiation doses on total chlorophyll content (A) and MDA (B) of the two freesia varieties. Lowercase letters on the same freesia variety represent statistically significant differences at *p* < 0.05. Error bars indicate the standard error of the mean for *N* = 3 independent experiments.

As irradiation dose increased, the total chlorophyll content of the two freesia varieties first increased and then decreased (Fig. 4A). The maximum total chlorophyll content was 0.56 mg kg^−^^1^ for the red freesia and 0.82 mg kg^−^^1^ for the purple freesia under 25-Gy treatment, which was significantly (*p* < 0.05) higher than that of the controls. Meanwhile, the MDA contents of the two freesia varieties increased with increasing irradiation dose (Fig. 4B). Interestingly, no significant differences in the MDA contents of the two freesia varieties were observed between the control and 25-Gy treatment, suggesting that low-level irradiation did not cause severe stress on the plants. The maximum MDA contents of the two freesia varieties were observed with 75-Gy treatment, which were significantly (*p* < 0.05) higher than those of the controls.

### Effects of different irradiation doses on the micro-morphology of leaf and pollen

#### Leaf micro-morphology

**Figure 5:**
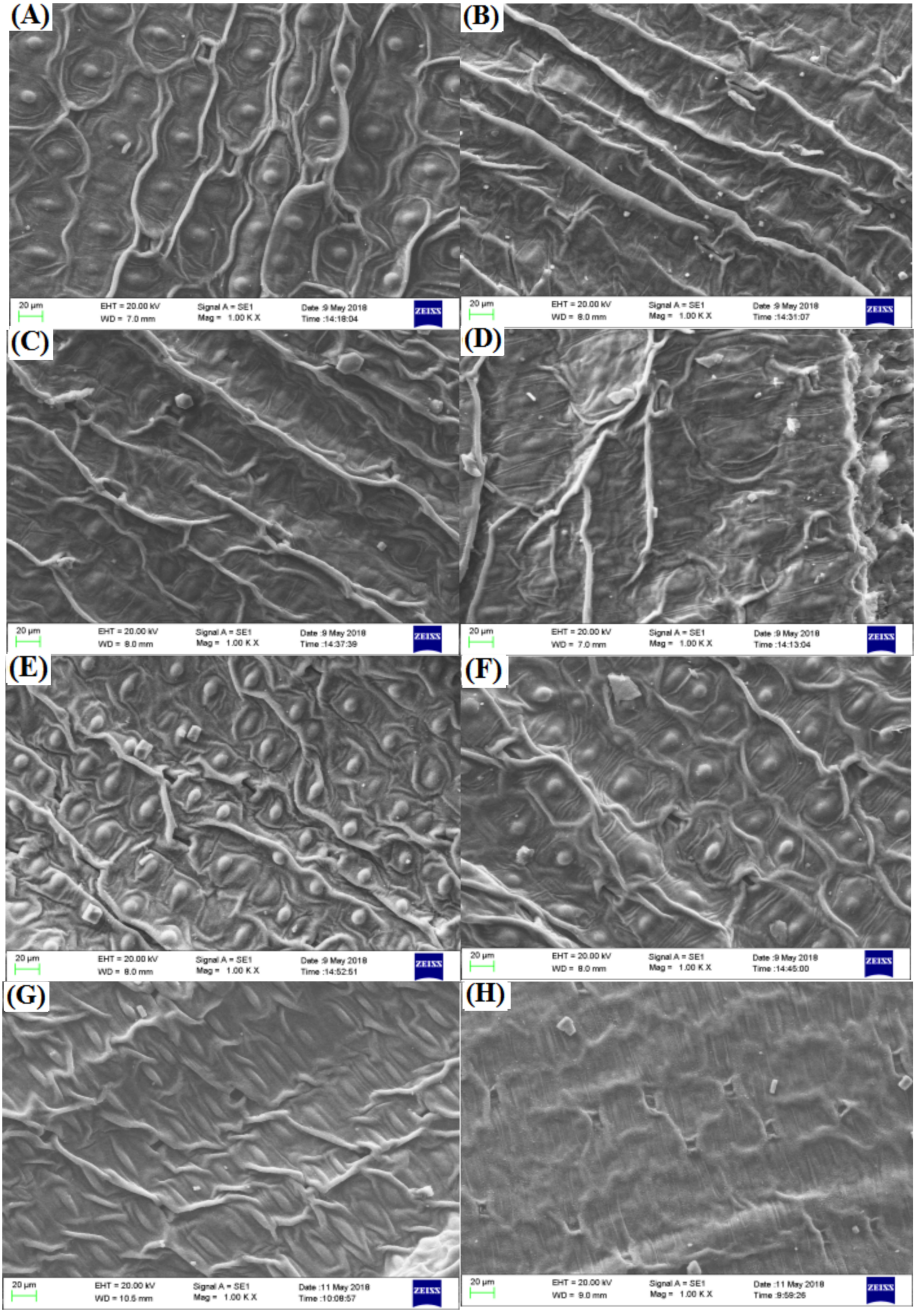
Influences of different irradiation doses on micro-morphology of the leaf epidermis. (A–D) (red freesia) indicates the CK (0 Gy), 25 Gy, 50 Gy, and 75 Gy treatment, respectively (20 µm). (E–H) (purple freesia) indicates the CK (0 Gy), 25 Gy, 50 Gy, and 75 Gy treatment, respectively (20 µm).

**Figure 6:**
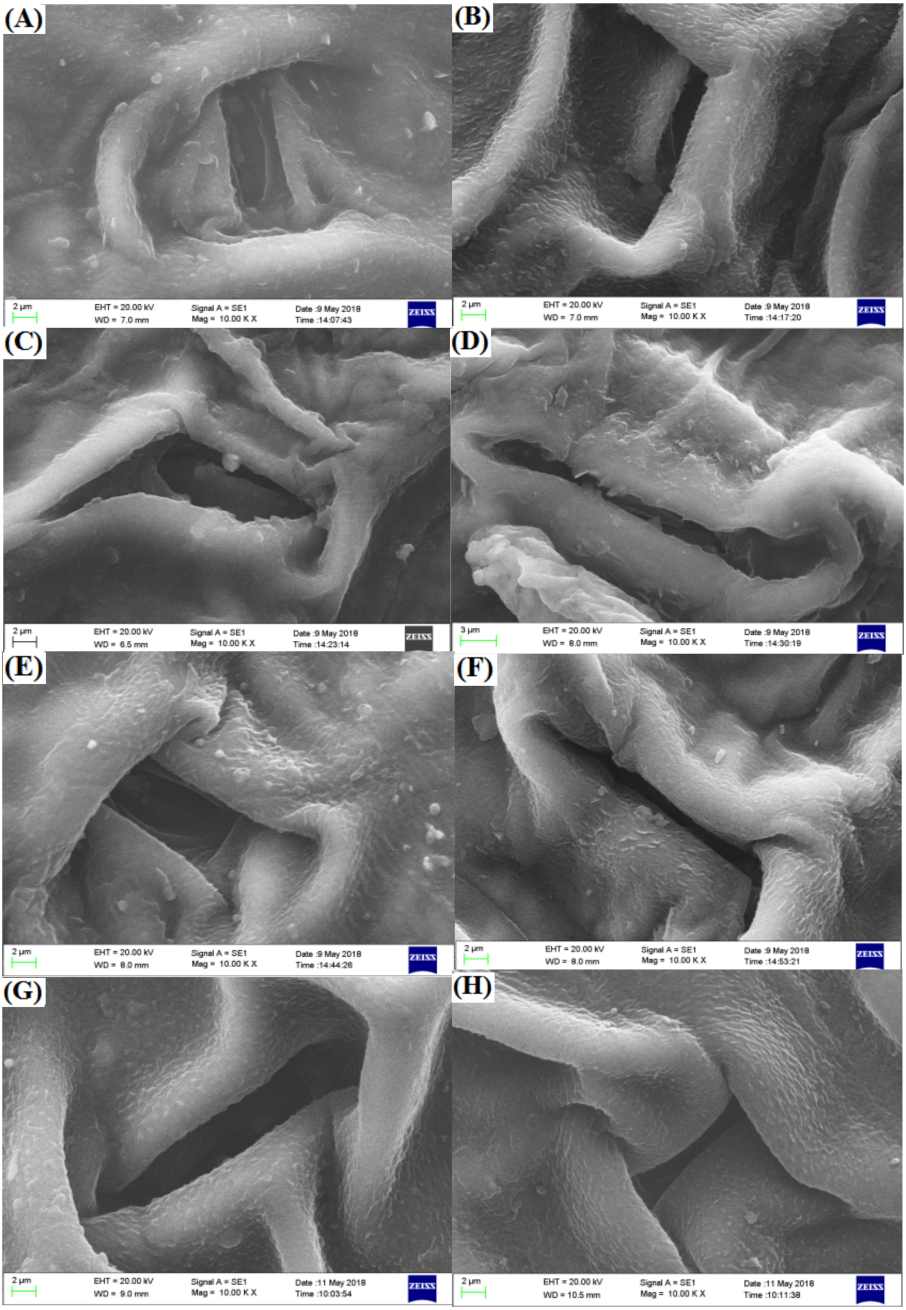
Influences of different irradiation doses on micro-morphology of the stomata. (A–D) (red freesia) indicates the CK (0-Gy), 25-Gy, 50-Gy, and 75-Gy treatment, respectively (2 -µm). (E–H) (the purple freesia) indicates the CK (0-Gy), 25-Gy, 50-Gy, and 75-Gy treatment, respectively (2-µm).

The spherical bulge of the two freesia varieties without irradiation treatment (Fig. 5) was regularly distributed on the leaf epidermis. However, the spherical bulge of the two freesia varieties gradually decreased with increasing irradiation dose, and the leaf epidermis following 75-Gy irradiation treatment was smoother compared with the controls. Meanwhile, the width of the stomata of the two freesia varieties gradually narrowed as the irradiation dose increased (Fig. 6). Moreover, shrinkage and deformation appeared in the stomata of the two freesia varieties under 75-Gy irradiation.

#### Pollen micro-morphology

**Figure 7:**
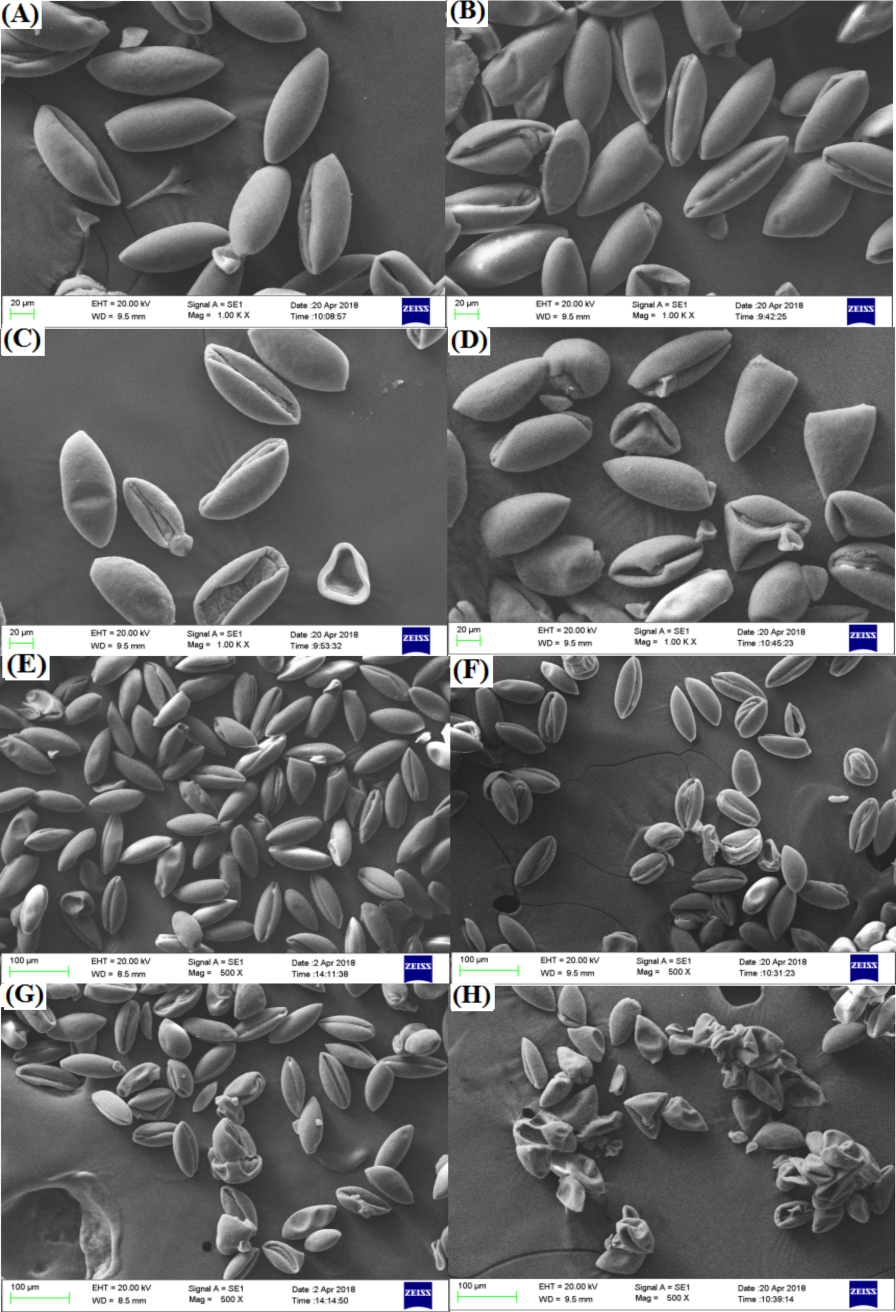
Influences of different radiation doses on micro-morphology of the pollen. (A–D) (the red freesia) indicates the CK (0-Gy), 25-Gy, 50-Gy, and 75-Gy treatment, respectively (20-µm). (E–H) (the purple freesia) indicates the CK (0-Gy), 25-Gy, 50-Gy, and 75-Gy treatment, respectively (100-µm).

Pollen of the two freesia varieties was approximately same size and uniform distribution, and its surface was smooth and plump (Fig. 7). The EBTTX irradiation treatments induced partial variation in the pollen of the two freesia varieties, and the variation was enhanced as the irradiation dose increased. Meanwhile, depressions and folds on the surface of the pollen were obviously observed under 75-Gy irradiation treatment, suggesting serious damage to the pollen.

## Discussion

### Plant growth

The results revealed that the growth of the two freesia varieties was inhibited by different irradiation doses, and high-dose irradiation exhibited stronger inhibition effects on plant growth compared with low-dose irradiation. The reason for this result is possibly due to the fact that irradiation can cause DNA breakage in plant cells, further leading to various types of damage to plant cell division and development, which constitutes the main damage to plant growth (Arena et al., 2014; Amirikhah et al., 2019). Compared with low-dose irradiation, a high irradiation dose even can induce cellular structural damage of bulbs of the two freesia varieties and decrease the nutrients and water uptake from soil to bulb, thus leading to severe inhibition of plant growth (Lung et al., 2015). Kim et al. (2019) also reported that the plant growth parameters such as germination rate, shoot length, root length, and fresh weight of creeping bentgrass (*Agrostis palustris Huds.*) significantly decreased with increasing irradiation doses of gamma rays and carbon ions compared with the control. Yadav, Singh & Singh (2019) also found that the plant height and leaf area of maize (*Zea mays* L.) were significantly decreased following 500-Gy gamma irradiation. Moreover, under different EBTTX irradiation doses, the survival rates of the two freesia varieties slightly increased as the growth time increased from 30 to 80 days, suggesting that freesia alleviates the irradiation toxicity to themselves with increasing growth time by developing antioxidant defence systems for scavenging the overproduction of free radicals induced by irradiation (Wang et al., 2018).

Median lethal dose (LD_50_) is an important indicator for analysing the sensitivity of plants to irradiation, which has been used as an important reference value to select the optimal irradiation dose in mutation breeding (Surakshitha, Soorianathasundaram & Ganesan, 2017). Our study showed that the LD_50_ was 54.28-Gy for the red freesia and 60.11-Gy for the purple freesia, suggesting that the red freesia is more sensitive to EBTTX irradiation than the purple freesia. Radiation affects the growth and development of plants by inducing heredity, and the process derived from gene regulation (Kadhimi et al., 2016). Meanwhile, the radiosensitivity of plants is related to genotype and varies with species (Albokari, Alzahrani & Alsalman, 2012). Thus, this could be attributed to the differences in the genotypes of the red freesia and the purple freesia. Moreover, we found that the two freesia varieties cannot survive under 100-Gy irradiation treatment. Similar phenomena have also been observed in studies of Moghaddam et al. (2011), who reported that the survival rate of *Centella asiatica* was less than 5% under 100-Gy irradiation treatment. The irradiation can interact with atoms and molecules to create free radicals in plant cells, which affects the plant cellular structure and metabolism, depending on the irradiation dosage (Gudkov et al., 2019).

Flowering is one of the most important and complex processes in the growth and development of ornamental plants, and it is highly sensitive to irradiation stress (Kryvokhyzha, Krutovsky & Rashydov, 2019). The results indicate that 75-Gy irradiation has a strong inhibitory effect on the flowering of the two freesia varieties. Kazama et al. (*2008*) also found that the flowering rate of *Arabidopsis thaliana* was decreased by 90.7% following heavy-ion beam irradiation at 150-Gy. Meanwhile, we found that pollen vigour continuously decreased with increasing irradiation dose from 0-100 Gy (Fig. 3C). Kundu et al. (2014) reported that the pollen vigour of the two citrus species (*C. limetta* and *C. sinensis*) significantly decreased following 400-Gy gamma ray treatment. Furthermore, we observed a phenomenon in the experiment in which 75-Gy EBTTX irradiation obviously delayed the initial flowering time of the two freesia varieties. Plants need to accumulate enough nutrients for effective reproductive growth during the vegetative growth stage (Santa-Maria, Moriconi & Oliferuk, 2015). If nutrition is not sufficient, plants have difficulty in completing flower development and seed maturation. Nevertheless, irradiation usually causes damage (mostly breaks) in DNA molecules directly by both transferring energy and generating free radicals, changing the physiological and biochemical processes of plants, and reducing the absorption of nutrients and water, thus inhibiting the reproductive growth of plants such as the flowering rate, flower number, and pollen vigour (Kazama et al., 2008; Arena, DeMicco & De Maio, 2014; Gudkov et al., 2019).

### Leaf chlorophyll content and lipid peroxidation

The leaf chlorophyll content can significantly affect the physiological and biochemical processes of plants, leading to changes in plant growth. In this study, 25-Gy EBTTX irradiation increased the total chlorophyll content, whereas 75-Gy EBTTX irradiation significantly restricted photosynthesis in the two freesia varieties. Chlorophyll content is an indicator of plant tolerance to irradiation, and assimilation plays an important role in photosynthesis. At low doses of radiation, plants enhanced assimilation and synthesized more secondary metabolites in response to stimuli (Sakalauskaité et al., 2013). Meanwhile, carotenoids in chlorophyll, including carotene and its oxygen-containing derivatives (such as lutein), involved in various antioxidant reactions and photosynthesis to activate defense systems and resist oxidative damage (Kim et al., 2004). When the levels of radiation increase above the maximum limit of the tolerance of the plants, the capabilities of the photosynthetic apparatus will be decreased by damaging the photosystem, thus resulting in a decrease in chlorophyll content (Perez-Ambrocio et al., 2018; Saha & Paul, 2019). In addition, high-dose irradiation can also indirectly affect photosynthesis by impairing the stomatal conductance and transpiration rate (Saha & Paul, 2019). Amirikhah et al. (2019) reported that low doses of gamma rays (15-krad) significantly increased the total chlorophyll content of tall fescue, but high doses of gamma rays (40-krad) inhibited chlorophyll synthesis of the plants.

MDA is a final decomposition product of lipid peroxidation, and the content of MDA in tissue is widely used to indirectly determine the physiological condition of the plant response against abiotic stress (Kumar et al., 2019; Chen et al., 2020). Our study showed that 75-Gy EBTTX irradiation significantly increased the MDA concentrations in the two freesia varieties, inferring clear oxidative damage under irradiation stress. Our findings are in agreement with Stajner, Popovic & Taski (2009), who found significantly enhanced MDA content under the application of 100-Gy gamma irradiation in soybean seeds (*Glycine max (Linn.)* Merr.). Moreover, the increase of MDA content by high irradiation doses has been reported in several plants, such as *Vigna sinensis* (El-Beltagi et al., 2013), *Lathyrus chrysanthus Boiss* (Beyaz et al., 2016), and *Arabidopsis thaliana* (Wang et al., 2018). When plants are irradiated by EBTTX, the intracellular water is decomposed into reactive oxygen species, which results in further damage to the plant cell membrane (Wang et al., 2018). Furthermore, the increasing trend of MDA content in the purple freesia following different irradiation doses is lower than that in the red freesia, suggesting that red freesia suffers stronger oxidative damage than purple freesia under radiation treatments.

### Leaf and pollen micro-morphology

Recently, several studies have reported the physiology and biochemistry of plants in response to ionizing irradiation (Kim et al., 2011; Wang et al., 2018). However, there is still little information about the effect of ionizing irradiation on the leaf and pollen micro-morphology of ornamental plants. In this study, we found that the spherical bulge of the leaf epidermis decreased with high irradiation doses, suggesting that the epidermal cells of the two freesia varieties were damaged by high irradiation doses. Compared with controls, the stomata of the two freesia varieties appeared to undergo shrinkage and deformation due to high doses of irradiation (75-Gy). These results are mainly attributed to the fact that the stomatal physiological functions of freesia leaves were seriously affected by high-dose irradiation (Kumar et al., 2019). The structure of the stomata of the two freesia varieties was changed under EBTTX irradiation treatment, thus reducing the carbon assimilation, water use, and plant growth and development (Mallick et al., 2016; Papanatsiou et al., 2019).

Pollen of ornamental plants is related to hereditary traits such as germination rate, flower colour, flowering stage, and flower type (Kundu et al., 2014). Our studies observed that the pollen of the two freesia varieties was seriously damaged by 75-Gy irradiation. High-energy irradiation induced DNA double-strand breakage in plant cells, and irregular or abnormal meiosis may cause significant changes in the pollen structure (Kurtar, 2009; Gudkov et al., 2019). Hirano et al. (2013) also indicated that the germination rate, pollen tube lengths and sperm cell formation of *Cyrtanthus mackenii* were significantly inhibited due to pollen damage in response to high-dose irradiation. The interior ultrastructure of pollen is very complex and contains sperm, cytoplasm and many organelles, which can ensure the normal reproduction of flowering plants (Yue et al., 2011). Thus, 75-Gy EBTTX irradiation is not suitable for mutation breeding of the two freesia varieties due to high levels of damage to the pollen.

## Conclusions

Overall, the present data suggested that the survival, growth and physiological status of the two freesia varieties are severely affected by a new radiation (EBTTX) stress. These results showed that radiation doses over 50-Gy (50–100 Gy) caused a significant inhibition in the two plant growth parameters, and photosynthesis, and an increase in the MDA content. Meanwhile, the two freesia varieties proved to be highly responsive to different doses of EBTTX irradiation based on all studied characters, and the sensitivity of the red freesia was always higher than of the purple freesia. Additionally, we speculated that the 54.28-Gy and 60.11-Gy was most suitable dose for mutation breeding of the red and purple freesia, respectively. Our results clearly indicated the effectiveness of EBTTX radiation for mutation breeding of the two freesia varieties; meanwhile, our findings could provide basic information for the future studies regarding the breeding programs of EBTTX irradiation in freesia and other ornamental plants. Furthermore, our future research should focus on the flower size and color of the two freesia varieties and the related molecular mechanisms under the EBTTX radiation.

##  Supplemental Information

10.7717/peerj.10742/supp-1 Supplemental Information 1 Original data

## Additional Information and Declarations

### Data Availability

The following information was supplied regarding data availability: The raw measurements are available as a Supplementary File.
